# Exploration of diarrhoea seasonality and its drivers in China

**DOI:** 10.1038/srep08241

**Published:** 2015-02-04

**Authors:** Zhiwei Xu, Wenbiao Hu, Yewu Zhang, Xiaofeng Wang, Maigeng Zhou, Hong Su, Cunrui Huang, Shilu Tong, Qing Guo

**Affiliations:** 1 School of Public Health and Social Work, Queensland University of Technology, Brisbane, Australia; 2 Institute of Health and Biomedical Innovation, Queensland University of Technology, Brisbane, Australia; 3 Center for Public Health Surveillance and Information Service, Chinese Center for Disease Control and Prevention, Beijing, China; 4 National Center for Chronic and Noncommunicable Disease Control and Prevention, Chinese Center for Disease Control and Prevention, Beijing, China; 5 School of Public Health, Anhui Medical University, Hefei, Anhui, China; 6 Centre for Environment and Population Health, School of Environment, Griffith University, Brisbane, Australia

## Abstract

This study investigated the diarrhoea seasonality and its potential drivers as well as potential opportunities for future diarrhoea control and prevention in China. Data on weekly infectious diarrhoea cases in 31 provinces of China from 2005 to 2012, and data on demographic and geographic characteristics, as well as climatic factors, were complied. A cosinor function combined with a Poisson regression was used to calculate the three seasonal parameters of diarrhoea in different provinces. Regression tree analysis was used to identify the predictors of diarrhoea seasonality. Diarrhoea cases in China showed a bimodal distribution. Diarrhoea in children <5 years was more likely to peak in fall-winter seasons, while diarrhoea in persons > = 5 years peaked in summer. Latitude was significantly associated with spatial pattern of diarrhoea seasonality, with peak and trough times occurring earlier at high latitudes (northern areas), and later at low latitudes (southern areas). The annual amplitudes of diarrhoea in persons > = 5 years increased with latitude (r = 0.62, *P*<0.001). Latitude 27.8° N and 38.65° N were the latitudinal thresholds for diarrhoea seasonality in China. Regional-specific diarrhoea control and prevention strategies may be optimal for China. More attention should be paid to diarrhoea in children <5 years during fall-winter seasons.

Despite remarkable progress in its control, diarrhoea remains a leading infectious cause of morbidity and mortality in low-income and middle-income countries, especially for children <5 years^1^,^2^,^3^,^4^. It is estimated that in 2010, worldwide, there were more than 1.7 billion episodes of diarrhoea in children <5 years, resulting in 700, 000 deaths in 2011^3^. China, together with other 14 countries, shoulder the heaviest burden of diarrhoea, accounting for 53% of total diarrhoea episodes and 56% of severe diarrhoea episodes in children aged <5 years^3^.

Exploring the seasonality of diarrhoea could reflect the relative predominance of its aetiological agents and shed new light on future vaccination programs. Further, understanding the determinants of seasonality could benefit the development of early warning systems. Existing research puts emphasis on exploring the seasonality of rotavirus diarrhoea and suggests that it varies with region and climate^5^,^6^,^7^,^8^. China is an economically, geographically and climatologically diverse country, in which the diversity of diarrhoea seasonality is not well unveiled.

After the 2003 severe acute respiratory syndrome (SARS) outbreak, Chinese Government has strengthened its public health disease system. The web-based real-time disease surveillance system-China Information System for Disease Control and Prevention (CISDCP), was built in 2004 to detect and respond to infectious disease outbreak^9^. Infectious diarrhoeal diseases, which have been reported to Chinese Center for Disease Control and Prevention (China CDC) through CISDCP in the past decade, were grouped into three classes: Class A (cholera); Class B (bacillary dysentery, typhoid and paratyphoid); and Class C (other infectious diarrhoea). The seasonality of Classes A and B, which may largely be determined by their predominant aetiological agents (bacterium *Vibrio* cholera, *shigellosis* and *salmonella*), has been extensively documented^10^,^11^. However, the seasonality of “other infectious diarrhoea” (Class C) remains unknown. In this study, we reviewed the data on “other infectious diarrhoea” in China from 2005–2012 obtained from CISDCP, aiming to characterize the seasonality of diarrhoea in China and identify its potential drivers, as well as explore potential opportunities for future diarrhoea control and prevention.

## Results

### Patterns of Seasonality

**Figure 1:**
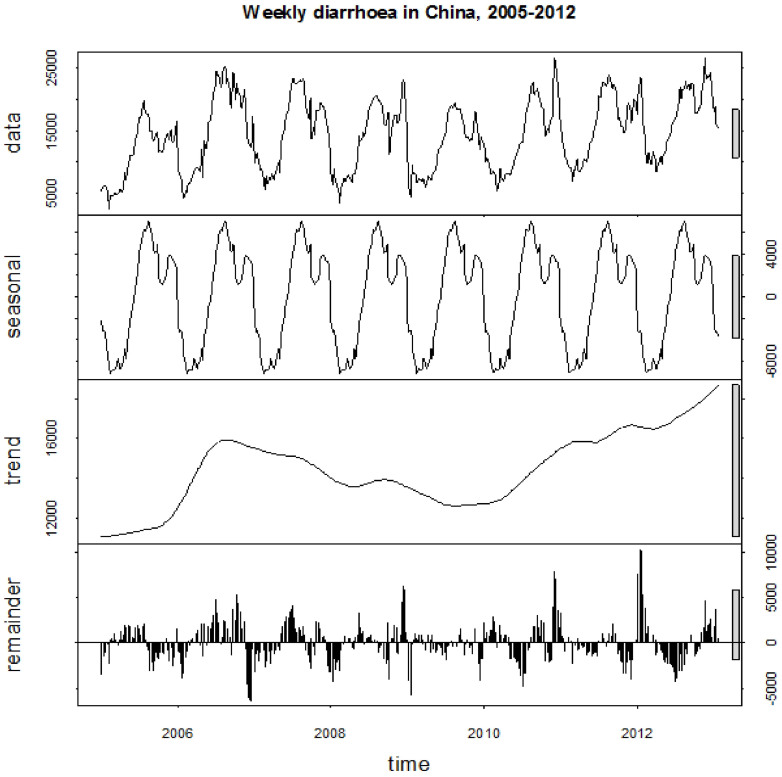
Temporal distribution of diarrhoea in China, from 2005 to 2012. Data: the raw time-series distribution of diarrhoea in China; seasonal: the seasonal trend decomposed from the raw data; trend: the long-term trend decomposed from the raw data; reminder: the reminding part which cannot be explained by seasonality or long-term trend.

**Figure 2:**
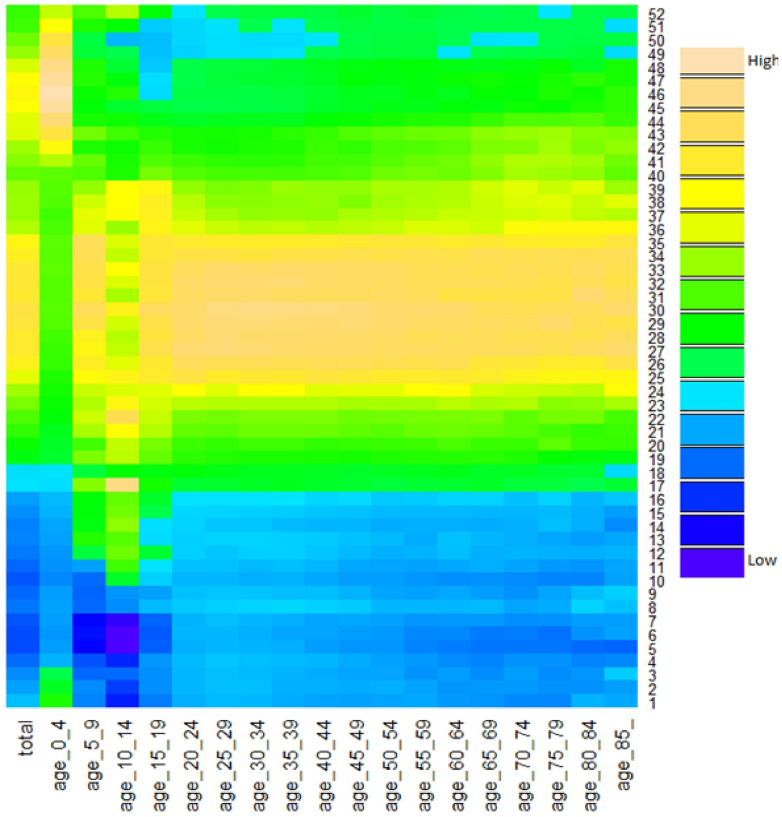
Time series of weekly diarrhoea cases in different age groups of China. Horizontal line: different age groups, from 0–4 years to > = 85 years; vertical line: week, from week 1 to week 52; colour palette: “High” refers to relatively higher number of diarrhoeal cases within each age group, and “Low” refers to relatively lower number of diarrhoeal cases within each age group.

There was a distinct seasonality in diarrhoea occurrence in the total population (Figure 1) and in each age group (Figures S1A–S1C (supplementary material)), and two peaks were observed every year. Specifically, diarrhoea in children <5 years peaked in fall-winter seasons, and diarrhoea in persons > = 5 years peaked in summer (Figure 2). Further, we found that the peak times in children (<15 years) varied greatly over time, while the peak times in adults (> = 15 years) was consistent across years (Figures S2A–S2F (supplementary material)).

**Figure 3:**
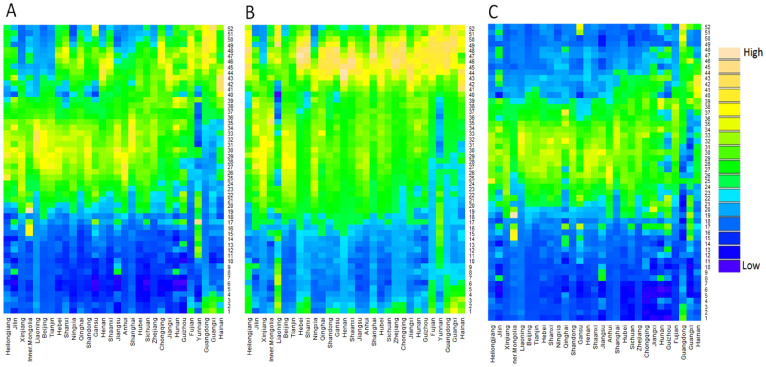
Heat maps of diarrhoea epidemiology data in China, from 2005 to 2012. (A) Time series of weekly diarrhoea cases in the total population, sorted by decreasing latitude from left to right. (B) Time series of weekly diarrhoea cases in children <5 years, sorted by decreasing latitude from left to right. (C) Time series of weekly diarrhoea cases in persons > = 5 years, sorted by decreasing latitude from left to right. Week 1 is the first week of January. Colour palette: “High” refers to relatively higher number of diarrhoeal cases within each province, and “Low” refers to relatively lower number of diarrhoeal cases within each province.

**Figure 4:**
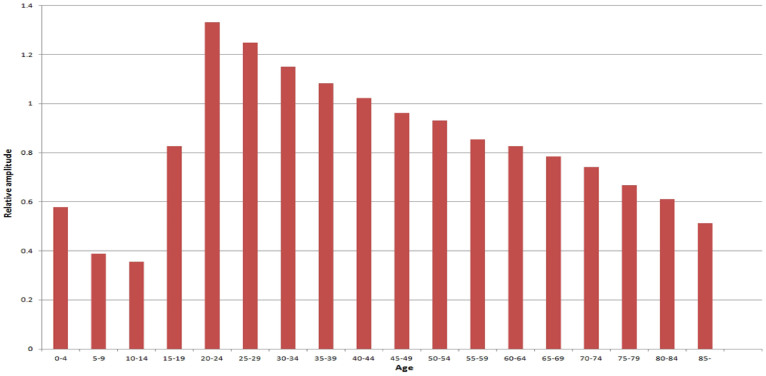
Diarrhoea amplitude in different age groups of China.

**Figure 5:**
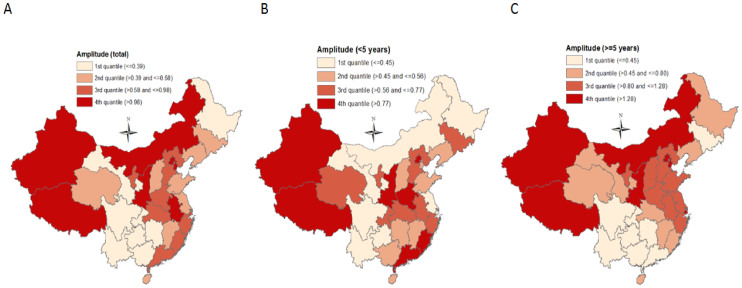
Estimates of diarrhoea amplitude in China, from 2005 to 2012. This figure was generated using ArcGIS 10 (ESRI, Redlands, CA, USA).

Figure 3 shows the seasonality of diarrhoea in different regions (listed by latitude), revealing that in both children <5 years and persons > = 5 years, diarrhoea at higher latitudes were more likely to be peaking in summer, and diarrhoea at lower latitudes were more likely to be peaking in fall-winter seasons (Figures 3A–C). Analysis by season (Figures S3A–3C (supplementary material)) reveals this pattern more clearly. In terms of the amplitude distribution by age and region (seasonal amplitude refers to the relative fluctuation of diarrhoea within a certain period of time, and it provides pivotal information on the possibility of diarrhoea outbreaks), we found that adults (> = 15 years) had much greater diarrhoea amplitudes than children (<15 years) (Figure 4), but the amplitude in persons > = 20 years declined progressively with increasing age, reaching its valley in elderly > = 85 years (Figure 4), and this decreasing trend was remarkably consistent across years (Figure S4 (supplementary material)). Figures 5A–C depicts the spatial pattern of diarrhoea amplitude, indicating that, in persons > = 5 years, diarrhoea amplitudes at higher latitudes were greater than lower latitudes.

### Drivers of seasonality

**Figure 6:**
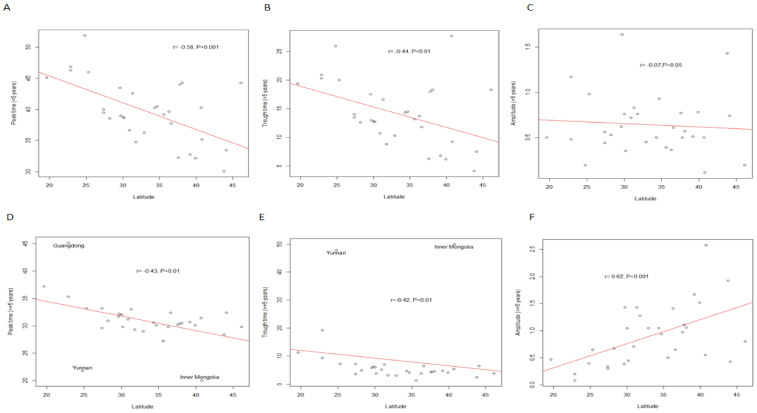
Scatter plots between latitude and diarrhoea seasonality parameters.

To better understand whether there was a statistically significant relationship between latitude and diarrhoea seasonal parameters (peak time, trough time and amplitude), we calculated the Spearman correlations between them, and found that diarrhoea peak time and trough time were negatively correlated with latitude (Figures 6A–D). No significant correlation was observed between amplitude in children <5 years and latitude, but amplitude in persons > = 5 years was positively correlated with latitude (r = 0.62, P<0.001) (Figures 6E–F).

**Figure 7:**
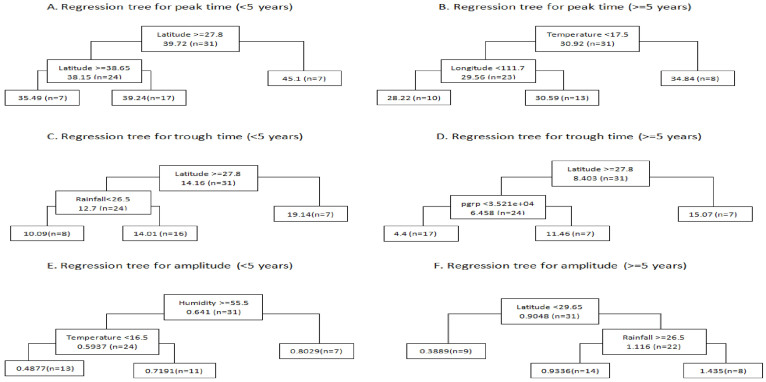
Predictors of diarrhoea seasonality in children <5 years and persons > = 5 years. The variables on the tree indicate the predictors of diarrhoea seasonality (e.g., Figure 7A implies that latitude is the primary predictor of diarrhoea peak time in children <5 years). The numbers on the tree refer to the thresholds of the predictors (e.g., Figure 7A suggests that, for the regions with latitude < 27.8°N, diarrhoea in children < 5 years are more likely to peak around week 45).

To identify the putative predictors of diarrhoea seasonality, we conducted regression tree analysis. Table 1 shows the summary statistics for the demographic, economic, and geographic characteristics, climatic factors, and diarrhoea seasonal parameters of the 31 provinces in China, which were included in the regression model. We found Latitude 27.8°N and Latitude 38.65°N were the latitudinal thresholds for diarrhoea seasonality in China (Figures 7A–D). Specifically, for provinces with latitude > = 27.8°N, diarrhoea in children <5 years reached its trough point between week 10 to 14, and jumped to the peak point between week 35 to 39, and diarrhoea in persons > = 5 years reached its trough point between week 4 to 11. For provinces with latitude < 27.8°N, diarrhoea in children <5 years reached its trough point in week 19, and jumped to the peak point in week 45, and diarrhoea in persons > = 5 years reached its trough point in week 15. Peak time in persons > = 5 years was associated with monthly mean temperature. Humidity and temperature may drive the distribution of amplitude in children <5 years, and latitude and rainfall were associated with amplitude in persons > = 5 years (Figures 7E–F).

### Epidemiological transmission zones

**Figure 8:**
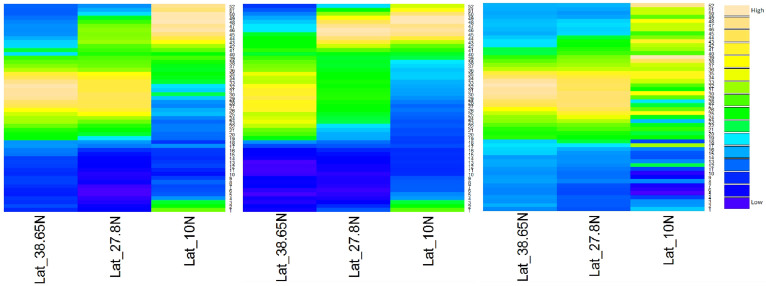
Heat maps of diarrhoea epidemiology data in three epidemiological regions, from 2005 to 2012.

After identifying the latitudinal threshold for diarrhoea peak and trough times, we plotted the heat maps accordingly (Figure 8A–C), and found that: At latitudes> = 38.65°N, diarrhoea in children <5 years and persons > = 5 years both peaked in summer; At latitudes > = 27.8 & <38.65°N, diarrhoea in children <5 years peaked in fall-winter seasons, but diarrhoea in persons > = 5 years peaked in summer; At latitudes <27.8°N, diarrhoea in children <5 years peaked in fall-winter seasons (slightly later than those between 27.8 to 38.65°N), while diarrhoea in persons > = 5 years did not have a distinct seasonality, peaking across the second half of each year.

## Discussion

The national surveillance data allows us to do this first comprehensive study concerning diarrhoea seasonality by age and geography in China. Diarrhoea in children <5 years normally peaked in fall-winter seasons, while diarrhoea in persons > = 5 years were more likely to peak in summer. Three epidemiological regions characterized by distinct diarrhoea seasonality were identified in this study: northern provinces (latitudes> = 38.65°N), intermediate provinces (latitudes> = 27.8°N & <38.65°N) and southern provinces (latitudes<27.8°N). Regression analysis indicated that mean temperature was predictive of diarrhoea peak time in persons > = 5 years, and relative humidity was linked to diarrhoea amplitude in children <5 years.

The core of seasonality in diarrhoea is related to temporal oscillations in the pathogenic agents and host susceptibility (fluctuations of neuroendocrine function and immune response^12^). The most intriguing finding of this study is the remarkable difference in the diarrhoea seasonality between children <5 years and persons > = 5 years. We found that diarrhoea in children <5 years appeared to peak in fall-winter seasons, which is in accord with findings in Brazil^13^, and this may partially be attributable to the fact that rotavirus is the predominant aetiology of diarrhoea in infants and young children^14^,^15^ and rotavirus favours low temperature^5^,^6^,^8^,^16^. Compared with infants and young children, persons > = 5 years in China are more likely to be attacked by bacterial pathogens^17^, possibly resulting in their summer peak time of diarrhoea^18^. Interestingly, we found diarrhoea seasonality (peak time, trough time and amplitude) in children <15 years varied over time, indicating a yearly-specific diarrhoea control and prevention strategy in children <15 years in China. Further, the consistent diarrhoea seasonality in adults > = 15 years across years we observed in this study may facilitate the development of future early warning systems focusing on diarrhoea control in adults.

In this study, we detected greater amplitudes of diarrhoea seasonality at higher latitudes among persons > = 5 years, implying that reduced winter sunlight and its potential effect on vitamin D deficiency might play a role in diarrhoea seasonality in persons > = 5 years. At northern latitudes, vitamin D deficiency is more common due to reduced ultraviolet light exposure^19^. At latitudes >40°N, even with adequate sun exposure, the dermal generation of vitamin D is negligible^20^,^21^, which may render the greater amplitudes of diarrhoea seasonality in persons > = 5 years by altering their immune response^22^. This finding should guide clinical and public health practice in those regions with high latitudes, and clinicians in those regions should be particularly made aware of the adverse impact of vitamin D insufficiency on diarrhoea and consider supplementation of vitamin D in persons with higher risk for diarrhoea. Moreover, we found amplitudes of diarrhoea seasonality in persons > = 15 years were much higher than children <15 years. This is striking because children shoulder majority of diarrhoea burden. Existing literature offers limited information on the mechanism explaining this finding, and we speculated that it may be because persons > = 15 years have had relatively recent diarrhoea compared with children <15 years^23^.

As reported in prior studies, though temperature, rainfall and relative humidity were associated with the occurrence of diarrhoea^5^,^6^,^7^,^8^, no climatic factor alone can fully capture the complexity of diarrhoea seasonality. However, in this study, we found climatic factors helped distinguish the peak time, trough time and amplitude between regions, which is pivotal for future diarrhoea control. Specifically, we found that 17.5°C was the temperature threshold for diarrhoea peak time in persons > = 5 years, with diarrhoea in those provinces with mean temperature below 17.5°C peaking earlier. Relative humidity was associated with seasonal amplitude of diarrhoea in children <5 years, and provinces with relative humidity below 55.5% had a greater seasonal amplitude of diarrhoea in children <5 years. It was also observed that 26.5 mm rainfall was the threshold for both trough time in children <5 years and amplitude in persons > = 5 years. For those regions with latitude > = 27.8°N and monthly average rainfall <26.5 mm, diarrhoea in children <5 years peaked the earliest, highlighting that public health sectors in these regions should take earlier precautionary measures to prevent infants and young children from being attacked by diarrhoea. Further, for the similar regions (latitude >29.65°N and monthly average rainfall < = 26.5 mm), the seasonal amplitude of diarrhoea in persons > = 5 years was the greatest. The identification of climatic factors associated with diarrhoea seasonality in this study will shed new light on the possible role of climate variation in the occurrence of diarrhoea.

Our large-scale analysis of diarrhoea seasonality identified three epidemiological regions: northern China where diarrhoea in children <5 years and persons > = 5 years both peaked in summer, mid-latitudes where diarrhoea in children <5 years peaked in fall-winter seasons and diarrhoea in persons > = 5 years peaked in summer, and southern China where diarrhoea in children <5 years peaked in fall-winter seasons and diarrhoea in persons > = 5 years peaked year-round. The three epidemiological zones indicate that diarrhoea seasonality in China may result from the interaction between climatic factors, behaviours likely to mix feces with food and water, and population immunity^3^,^7^. The seasonal patterns of diarrhoea at low latitudes (<27.8°N) in children <5 years and persons > = 5 years we found were not consistent with prior studies^6^,^7^,^8^,^24^. Previous research mainly focused on rotavirus seasonality in children <5 years and found that rotavirus persists year-round in tropical areas (<24°N) and peaks from autumn to spring (cold and dry seasons) in temperate areas^6^. Information on diarrhoea seasonality in adults is scarce in existing literature, and one study looking at the rotavirus diarrhoea in Japan did not find a significant seasonality^25^. The motivation of this study is to assist on-going diarrhoea surveillance and the epidemiological regions we identified have important implications for development of early warning system. Specifically, in northern China, diarrhoea preventive measures should be taken earlier (before summer); in intermediate latitudes, more attention should be paid to diarrhoea in children <5 years during fall-winter seasons, and diarrhoea control may focus more on persons> = 5 years in summer, and in southern China, diarrhoea control should also focus on children <5 years during fall-winter seasons while diarrhoea surveillance in persons > = 5 years should be strengthened, given its non-distinct seasonality.

This is the first study to explore the seasonality of diarrhoea in China at the national level. The remarkably different diarrhoea seasonality in children <5 years and persons > = 5 years we found will be useful for designing and implementing future diarrhoea control and prevention strategies. The three epidemiological zones we identified will guide the development of targeted early warning systems and implementation of future risk management efforts. Further, the potential drivers of diarrhoea seasonality we identified will facilitate future studies assessing the impact of social-environmental change on diarrhoea in China. Several limitations should also be acknowledged. First, these diarrhoea cases were not all lab-confirmed. Second, most diarrhoea cases may be undetected as people with mild symptoms may not seek medical care. Third, access to health care may vary across provinces, and we cannot rule out surveillance bias in some areas such as Tibet and Xinjiang. Finally, we did not have data on the pathogenic agents, which restricts us to examine the seasonality of specific pathogens and give more evidence-based implications for future vaccination programs.

In conclusion, our study unfolds the striking difference in diarrhoea seasonality between children <5 years and persons > = 5 years. The distinct geographic patterns of diarrhoea, which may be impacted by climatic factors, were also unveiled. Future research should focus more on elucidating the impact of social-environmental changes on diarrhoea in different epidemiological zones and mechanisms why diarrhoea seasonality differs greatly across different age groups. Our work has practical implications for the development of early warning systems targeting different population in different regions.

## Methods

### Data collection

The territory of China lies between latitudes 18° and 54°N, and longitudes 73° and 135°E. China's climate is mainly dominated by dry seasons and wet monsoons, and it is diverse from region to region due to the complex topography of China. Table 1 shows the summary statistics for the demographic, economic, and geographic characteristics as well as climatic factors of 31 Chinese provinces. Infectious diarrhoea data from 1^st^ January 2005 to 31^st^ December 2012 were collected from CISDCP. CISDCP is the reporting system of nationally notifiable infectious diseases and public health emergencies throughout China^26^. Diarrhoea cases were those who have three or more loose or liquid stools per day diagnosed in the hospital or the local CDC. In this study, we used the weekly reports of diarrhoea cases from CISDCP. Information on the age and residential address of the diarrhoea patients was obtained. Ethical approval was obtained from the Research Institutional Review Board of Public Health of Shandong University (China) prior to the data being collected. Patient information was de-identified and thus no written informed consent was obtained.

Demographic, economic, geographic and climate information in all 31 provinces from 2005 to 2012, including population, per capita gross regional product (PGRP), latitude, longitude, monthly average mean temperature, monthly average relative humidity, and monthly average rainfall, were collected from China Statistical Yearbook^27^ to identify the putative drivers of diarrhoea seasonality.

### Data analysis

A seasonal decomposition analysis was conducted to assess whether there was a distinct seasonality in diarrhoea occurrence^28^. In this analysis, diarrhoea time-series was decomposed into seasonality, long-term trend, and irregular factors^23^. Heat maps were created to present the peak and trough times of diarrhoea in each age group and province. A cosinor function combined with Poisson regression was used to quantify the seasonal parameters of diarrhoea (ie., peak time, trough time and annual amplitude)^29^. The annual amplitude was expressed as a proportion of mean diarrhoea cases to facilitate the comparisons between different age groups and different regions. To identify the putative drivers of diarrhoea seasonality, a classification and regression tree (CART) analysis was conducted^30^. CART model is based on simple splits of the data and does not require the assumptions such as the existence of linear regression among and variables and homoscedasticity in variance. CART model was chosen in this study mainly because the potential drivers of diarrhoea seasonality (e.g., latitude and temperature) are highly correlated with each other and CART can potentially better accommodate complex interactions between variables since they avoid some of the assumptions associated with linear regression^31^. More importantly, CART model can identify the thresholds for the independent variables in the regression. Visual maps for the spatial distributions of diarrhoea incidence and annual amplitude were created using ArcGIS version 9.3 (ESRI Inc., Redlands, CA, USA). All the remaining analysis was conducted using the R statistical environment (version 2.15.3), with the “season” package (version 0.3-3) to conduct the cosinor analysis and “rpart” package to conduct the regression tree analysis.

## Author Contributions

Z.X., W.H., Q.G. and S.T. designed the study and collected the data, Z.X. and W.H. analysed the data and drafted the manuscript, W.H., Y.Z., X.W., M.Z., H.S., C.H., S.T. and Q.G. revised the manuscript.

## Supplementary Material

Supplementary Information Supplementary information

## Figures and Tables

**Table 1:** Summary statistics for demographic and geographic characteristics, climatic factors, and diarrhoea seasonal parameters of the 31 provinces in China

Region	Latitude	Longitude	PGRP^a^	Temperature (°C)	Humidity (%)	Rainfall (mm)	Peakt^b^	Peakc^c^	Peaka^d^	Lowt^e^	Lowc^f^	Lowa^g^	Amplitudet^h^	Amplitudec^i^	Amplitudea^j^
Anhui	31.8	117.5	17121.6	17	74	32	30.7	34.8	29.3	4.7	8.8	3.2	1.01	0.76	1.28
Beijing	39.9	116.4	66148.96	13	51	23	30.5	32.2	30.1	4.5	6.2	4.1	1.31	0.78	1.52
Chongqing	29.6	106.6	23416.73	19	78	35	39.3	43.5	31.7	13.3	17.5	5.7	0.39	0.62	0.39
Fujian	25.3	118.8	33568.41	21	70	33	42.5	46	33.2	16.5	20	7.2	0.62	0.98	0.65
Gansu	35.6	104.7	13814.94	9	54	27	32.3	39.2	27.2	6.3	13.2	1.2	0.33	0.39	0.5
Guangdong	22.9	113.4	39037.68	23	72	33	46.3	46.3	45.2	20.3	20.3	19.2	0.76	1.17	0.2
Guangxi	22.9	108.4	16876.84	21	76	35	45.8	46.9	35.3	19.8	20.9	9.3	0.33	0.48	0.08
Guizhou	27.4	106.8	11215.5	15	76	34	34.9	39.5	29.6	8.9	13.5	3.6	0.3	0.44	0.3
Hainan	19.6	110.1	20029.68	24	80	35	41.4	45.1	37.2	15.4	19.4	11.2	0.4	0.5	0.47
Hebei	38.1	115.8	24703.15	14	56	26	33.7	44.3	30.5	7.7	18.3	4.5	0.59	0.57	1.06
Heilongjiang	46.1	126.2	23631.59	6	64	27	33.3	44.3	29.8	7.3	18.3	3.8	0.28	0.19	0.8
Henan	34.7	113.1	20609.16	16	58	28	35.4	40.5	30.1	9.4	14.5	4.1	0.71	0.93	0.94
Hubei	30.9	112.6	23063.65	17	72	33	34	36.7	31.2	8	10.7	5.2	0.67	0.72	0.71
Hunan	27.4	113	20507.28	18	72	33	37.2	40	33.2	11.2	14	7.2	0.39	0.56	0.34
Mongolia	40.8	110.8	38372.48	8	46	21	28.4	35.2	20	2.4	9.2	50	1.68	0.11	2.58
Jiangsu	32.9	118.6	44292.88	16	71	33	31.3	36.3	29	5.3	10.3	3	0.51	0.45	1.05
Jiangxi	28.2	115.3	17904.59	19	69	34	36.9	38.6	30.9	10.9	12.6	4.9	0.56	0.53	0.67
Jilin	44.1	125.4	26497.15	6	61	27	33.2	33.5	32.4	7.2	7.5	6.4	0.58	0.74	0.43
Liaoning	40.7	122.6	35434.71	8	67	27	31.5	40.3	31.4	5.5	27.7	5.4	0.5	0.5	0.55
Ningxia	37.6	106	21880.91	10	51	23	32.2	32.3	30.2	6.2	6.3	4.2	0.71	0.77	0.97
Qinghai	36.6	101.8	20122.19	6	56	24	36.1	37.8	32.4	10.1	11.8	6.4	0.55	0.61	0.65
Shaanxi	34.3	108.8	22477.03	15	63	31	36.2	40.3	30.6	10.2	14.3	4.6	0.53	0.5	1.05
Shandong	36.3	118.4	34993.01	15	56	28	32.1	39.7	29.8	6.1	13.7	3.8	0.71	0.36	1.41
Shanghai	31.3	121.5	67924.76	17	69	31	33.9	42.6	33	7.9	16.6	7	1.22	0.83	1.43
Shanxi	37.8	112.8	22389.53	11	53	25	35.5	44	30.4	9.5	18	4.4	0.48	0.5	1.11
Sichuan	30.2	104	17798.11	16	76	30	33.5	38.7	29.8	7.5	12.7	3.8	0.35	0.35	0.45
Tianjin	39.2	117.2	62335.01	13	58	26	30.9	32.8	30.7	4.9	6.8	4.7	1.41	0.51	1.67
Tibet	29.7	91.1	11502.42	10	34	35	35.2	39	32.2	9.2	13	6.2	1.43	1.64	1.4
Xinjiang	43.8	87.6	21660.51	8	55	25	29.3	30.1	28.4	3.3	4.1	2.4	1.64	1.43	1.92
Yunan	24.8	103	13821.13	16	69	31	17.1	51.9	21.8	34.1	25.9	47.8	0.05	0.19	0.4
Zhejiang	30	120.4	44229.7	18	71	30	35	38.8	32	9	12.8	6	0.98	0.76	1.04

^a^Per capita gross regional product;

^b^Peak time in the total population;

^c^Peak time in children <5 years;

^d^Peak time in persons > = 5 years;

^e^Trough time in the total population;

^f^Trough time in children <5 years;

^g^Trough time in persons > = 5 years;

^h^Amplitude in the total population;

^i^Amplitude in children <5 years;

^j^Amplitude in persons > = 5 years.
