# Supplementary material for: Exploration of diarrhoea seasonality and its drivers in China
Source: Sci Rep. 2015 Feb 4;5:8241. doi: 10.1038/srep08241 (PMC4316158; doi:10.1038/srep08241)
Supplement: Supplementary Information [file srep08241-s1.pdf]

Appendix

Exploration of diarrhoea seasonality and its drivers in China

Zhiwei Xu[1,2†], Wenbiao Hu[1,2†], Yewu Zhang[3], Xiaofeng Wang[3], Maigeng Zhou[4], Hong Su[5], Cunrui Huang[6], Shilu Tong[1,2*], Qing Guo[3*]

1 School of Public Health and Social Work, Queensland University of Technology, Brisbane, Australia.

2 Institute of Health and Biomedical Innovation, Queensland University of Technology, Brisbane, Australia.

3 Center for Public Health Surveillance and Information Service, Chinese Center for Disease Control and Prevention, Beijing, China

4 National Center for Chronic and Noncommunicable Disease Control and Prevention, Chinese Center for Disease Control and Prevention, Beijing, China.

5 School of Public Health, Anhui Medical University, Hefei, Anhui, China.

6 Centre for Environment and Population Health, School of Environment, Griffith University, Brisbane, Australia

[†] These authors equally contributed to the manuscript.

Correspondence and requests for materials should be addressed to:

Ms. Qing Guo, Center for Public Health Surveillance and Information Service, Chinese Center for Disease Control and Prevention, 27 Nanwei Road, Xicheng District, Beijing, 100050, China. Email address: guoqing@chinacdc.cn

Prof. Shilu Tong, School of Public Health and Social Work & Institute of Health and Biomedical Innovation, Queensland University of Technology, Kelvin Grove, QLD. 4059, Australia. Email address: s.tong@qut.edu.au

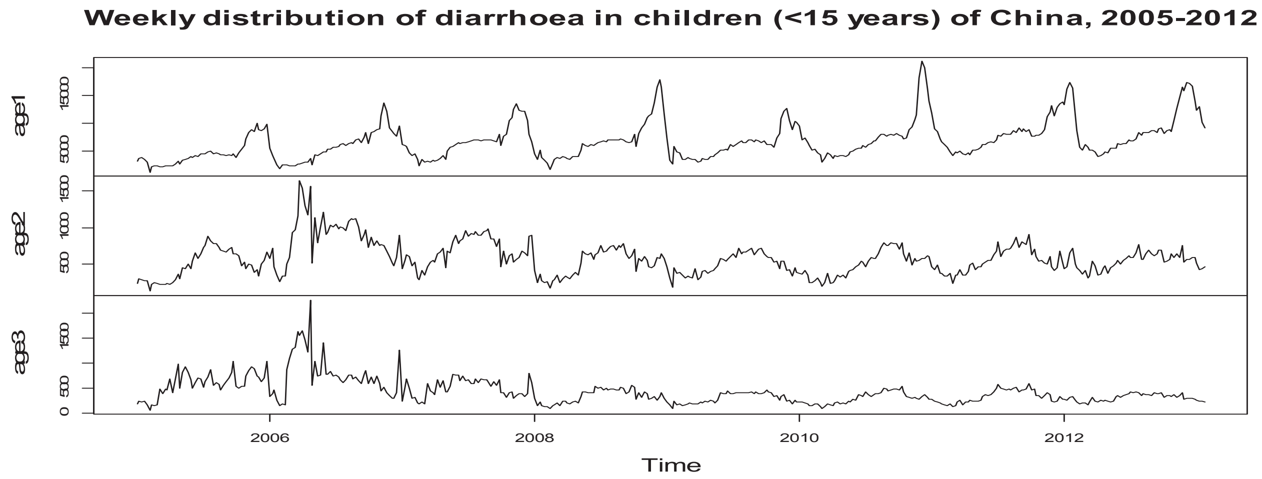

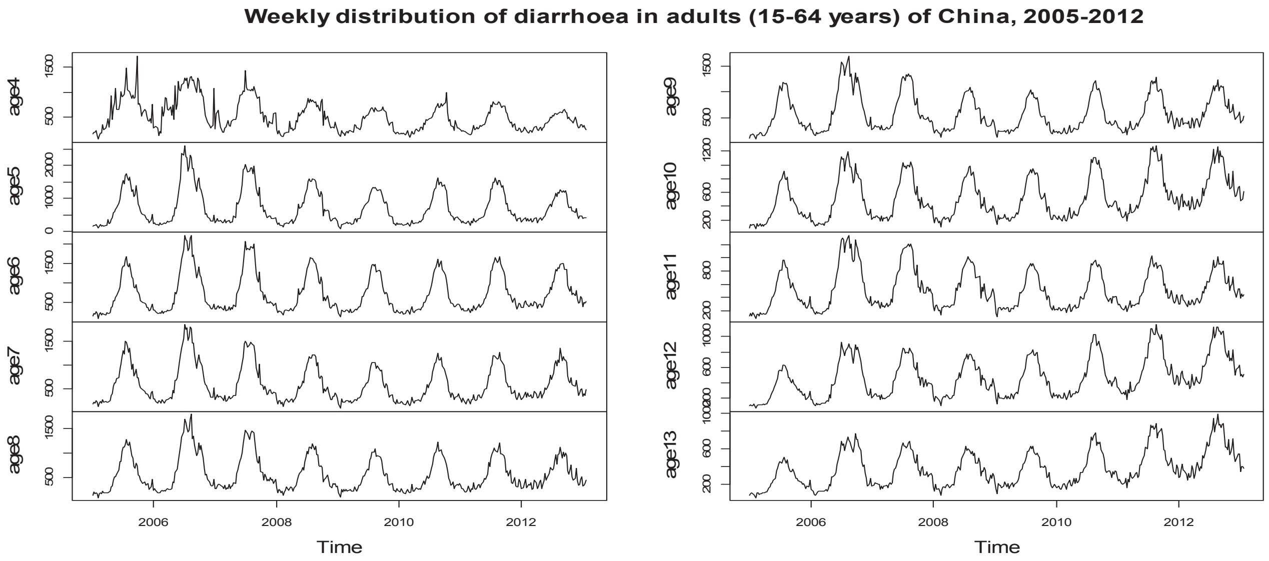

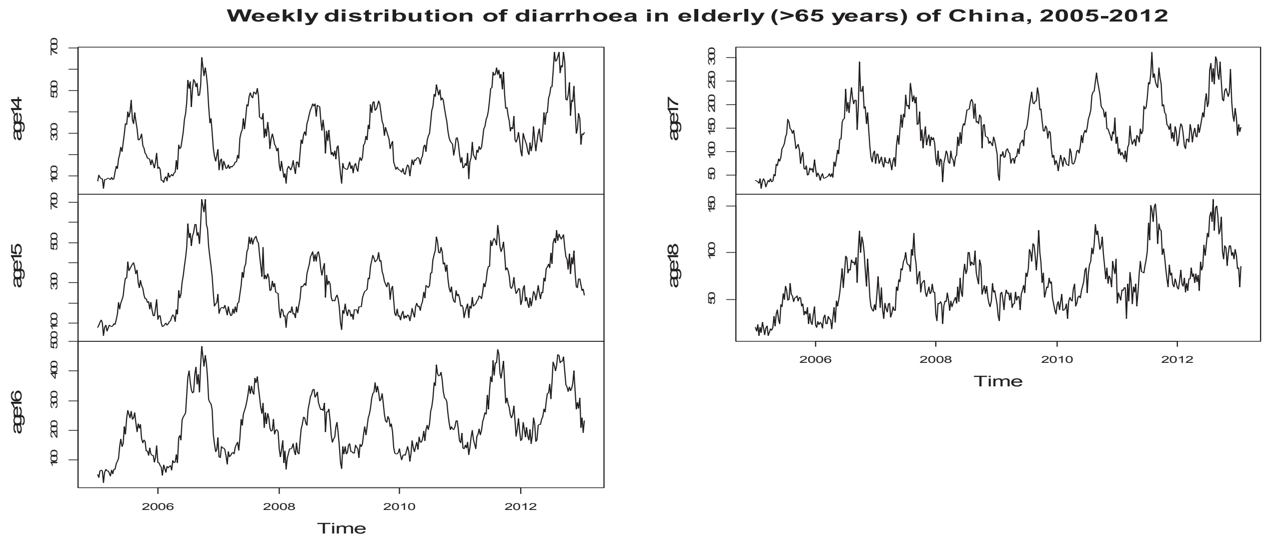

Figure S1. Temporal distributions of diarrhoea by age in China, from 2005 to 2012. Each group has an age range of five. Age 1: 0-4 years, age 17: 80-84 years, and age 18: >=85 years.

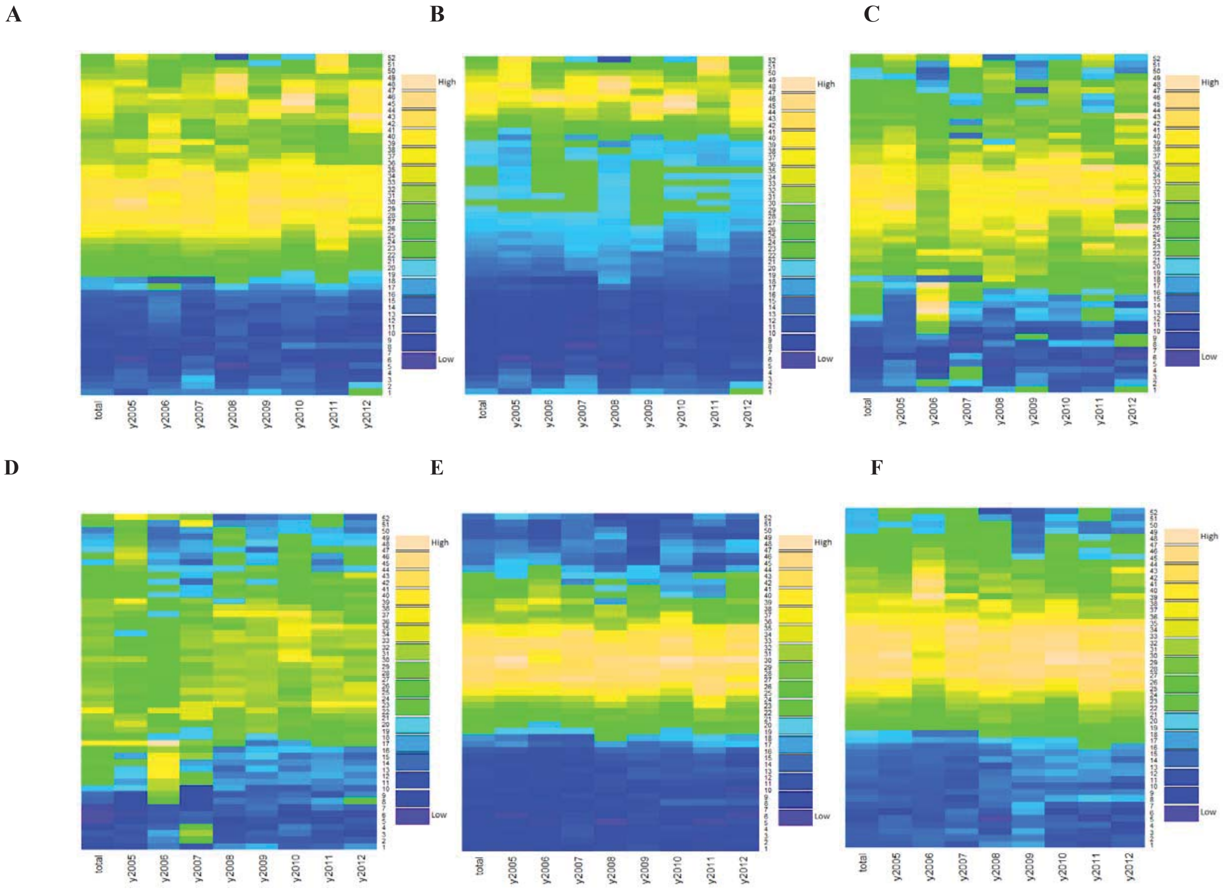

Figure S2. Change over time in weekly distribution of diarrhoea by age. A: Total population, B: 0-4 years, C: 5-9 years, D: 10-14 years, E: 15-64 years, F: >=65 years

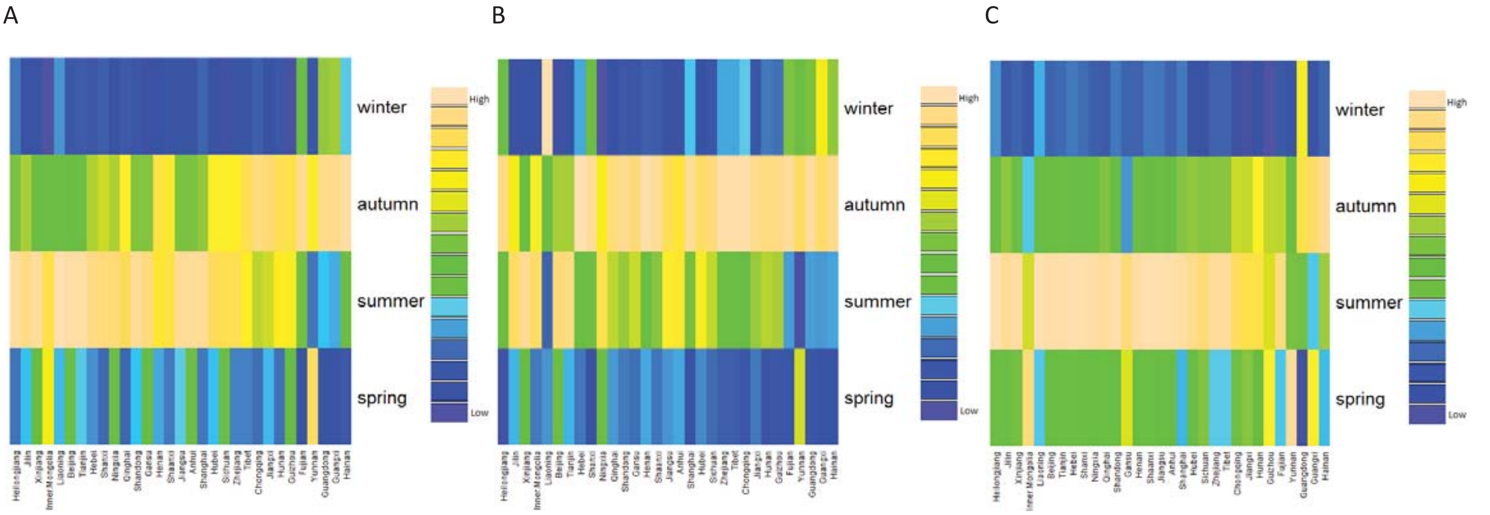

Figure S3. Heat maps of diarrhoea epidemiology data in China, from 2005 to 2012. (A) Diarrhoea cases in the total population by season, sorted by decreasing latitude from left to right. (B) Diarrhoea cases in children <5 years by season, sorted by decreasing latitude from left to right. (C) Diarrhoea cases in the total population in persons >=5 years by season, sorted by decreasing latitude from left to right.

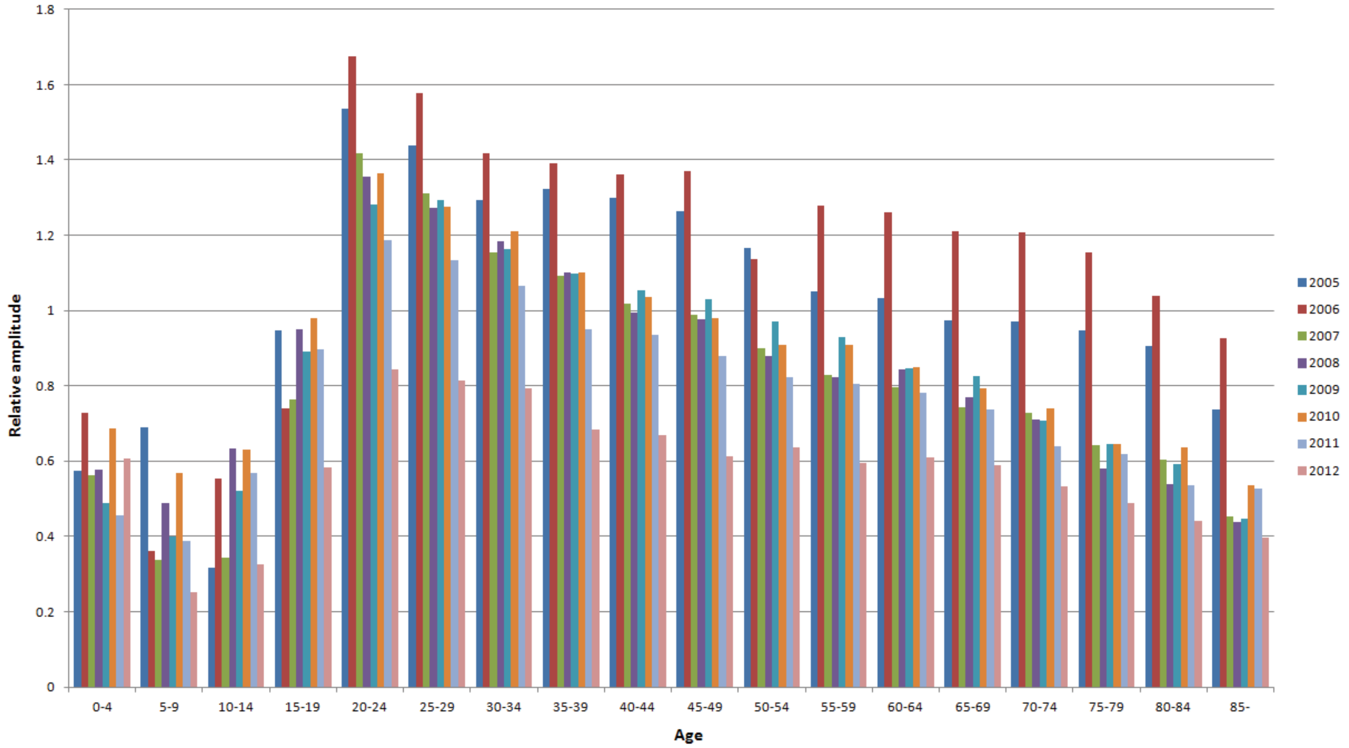

Figure S4. Change over time in the amplitude of diarrhoea seasonality by age.
